# Infarct size and left ventricular remodelling after preventive percutaneous coronary intervention

**DOI:** 10.1136/heartjnl-2015-308660

**Published:** 2016-08-08

**Authors:** Kenneth Mangion, David Carrick, Barry W Hennigan, Alexander R Payne, John McClure, Maureen Mason, Rajiv Das, Rebecca Wilson, Richard J Edwards, Mark C Petrie, Margaret McEntegart, Hany Eteiba, Keith G Oldroyd, Colin Berry

**Affiliations:** 1 BHF Glasgow Cardiovascular Research Centre, University of Glasgow, Glasgow, UK; 2 West of Scotland Heart and Lung Centre, Golden Jubilee National Hospital, Dunbartonshire, UK; 3 Therapeutics and Cardiac Research Team, Freeman Hospital, Newcastle upon Tyne, UK

## Abstract

**Objective:**

We hypothesised that, compared with culprit-only primary percutaneous coronary intervention (PCI), additional preventive PCI in selected patients with ST-elevation myocardial infarction with multivessel disease would not be associated with iatrogenic myocardial infarction, and would be associated with reductions in left ventricular (LV) volumes in the longer term.

**Methods:**

In the preventive angioplasty in myocardial infarction trial (PRAMI; ISRCTN73028481), cardiac magnetic resonance (CMR) was prespecified in two centres and performed (median, IQR) 3 (1, 5) and 209 (189, 957) days after primary PCI.

**Results:**

From 219 enrolled patients in two sites, 84% underwent CMR. 42 (50%) were randomised to culprit-artery-only PCI and 42 (50%) were randomised to preventive PCI. Follow-up CMR scans were available in 72 (86%) patients. There were two (4.8%) cases of procedure-related myocardial infarction in the preventive PCI group. The culprit-artery-only group had a higher proportion of anterior myocardial infarctions (MIs) (55% vs 24%). Infarct sizes (% LV mass) at baseline and follow-up were similar. At follow-up, there was no difference in LV ejection fraction (%, median (IQR), (culprit-artery-only PCI vs preventive PCI) 51.7 (42.9, 60.2) vs 54.4 (49.3, 62.8), p=0.23), LV end-diastolic volume (mL/m^2^, 69.3 (59.4, 79.9) vs 66.1 (54.7, 73.7), p=0.48) and LV end-systolic volume (mL/m^2^, 31.8 (24.4, 43.0) vs 30.7 (23.0, 36.3), p=0.20). Non-culprit angiographic lesions had low-risk Syntax scores and 47% had non-complex characteristics.

**Conclusions:**

Compared with culprit-only PCI, non-infarct-artery MI in the preventive PCI strategy was uncommon and LV volumes and ejection fraction were similar.

## Introduction

Patients with acute ST-elevation myocardial infarction (STEMI) and multivessel coronary artery disease have an increased risk of adverse outcomes,^1^ ^2^ however, the optimal management of non-culprit lesions is controversial. Following primary percutaneous coronary intervention (PCI), additional PCI of non-culprit-artery lesions might prevent recurrent ischaemia and adverse cardiac events.^1^ ^2^ On the other hand, it may cause complications, including iatrogenic myocardial infarction secondary to the PCI procedure through coronary microembolisation leading to a larger overall infarct burden.^3^ ^4^ Evidence of harm associated with non-culprit-artery PCI in large registries^5^ ^6^ and systematic reviews^7^ underpins the caution in current guideline recommendations regarding routine PCI of non-culprit-artery lesions during primary PCI.^8–10^

In the randomised trial of preventive angioplasty in myocardial infarction (PRAMI; ISRCTN73028481^11^), immediate preventive PCI of angiographically significant non-culprit-artery stenoses in 465 patients with STEMI and multivessel disease reduced the incidence of the composite primary outcome (cardiac death, non-fatal myocardial infarction (MI), refractory angina) by 14% at 2 years.

Similar results have recently been reported in the complete versus culprit lesion-only PRimary PCI trial (CvLPRIT) (ISRCTN70913605) trial,^12^ but in the DANAMI-3-PRIMULTI trial the benefit of complete revascularisation was driven by significantly fewer repeat revascularisations, because all-cause mortality and non-fatal reinfarction did not differ between groups.^13^ Taken together, the relative risk reduction of the primary composite outcome in these three trials ranges from 45% to 65%.^11–13^ On the other hand, these trials were not powered to assess cardiac mortality and the performance and timing of non-culprit vessel PCI after primary PCI remains an open and controversial question. In 203 patients in the culprit cardiac magnetic resonance (CMR) substudy there was a statistically significant increase in non-infarct-related artery MI in the complete revascularisation group but total infarct size was not significantly different compared with an infarct related artery (IRA) only strategy.^14^ Specifically, questions still remain about the risk of iatrogenic MI secondary to the additional PCI procedures increasing infarct size overall, and the associations between non-culprit PCI and left ventricular (LV) ejection fraction and volumes in the longer term.

We hypothesised that, compared with culprit-only primary PCI, additional preventive PCI in selected STEMI patients with multivessel disease would (1) not be associated with iatrogenic myocardial infarction secondary to the preventive PCI procedures and (2) would have a similar infarct size. CMR is the reference diagnostic imaging method for the assessment of infarct size^15^ and LV volumes.^16^ In a prespecified CMR substudy, we prospectively assessed infarct size and LV volumes. CMR has potential to be clinically useful for the assessment of procedure-related MI, which cannot otherwise be reliably detected using a troponin elevation or the electrocardiogram in the setting of acute STEMI.

## Methods

### Setting

The PRAMI CMR substudy took place in the Golden Jubilee National Hospital, Clydebank and the Freeman Hospital, Newcastle. Both of these hospitals are regional cardiac centres. Acute STEMI management follows contemporary guidelines. Aspiration thrombectomy, direct stenting, antithrombotic drugs and other therapies were administered according to clinical judgement and in line with the guidelines.^8–10^ Further details are available in the online supplement.

10.1136/heartjnl-2015-308660.supp1 Supplementary data

### CMR acquisition

CMR scans were performed during the index hospitalisation within (median, IQR) 3 (1, 5) days post-randomisation and repeated 209 (189, 957) days after primary PCI.

### CMR methods

#### CMR image acquisition

Information about MRI acquisition is provided in the online supplement.

#### CMR image analysis

Image analysis was performed using a core laboratory with dedicated CMR software (Siemens Syngo VE32D and Argus, Erlangen, Germany). CMR data were anonymised and then analysed in a random order by a CMR-trained cardiologist (KM) who was blinded to the treatment group assignment and all other clinical and health outcome data. Image quality was assessed using Likert scale quality scores.

#### Infarct definition and size

The myocardial mass of late gadolinium was quantified using computer-assisted planimetry as a hyperintense region over five SDs the signal intensity of remote (see online supplement).

#### Adverse remodelling

Adverse remodelling was defined as an increase in LV end-diastolic volume and end-systolic volume ≥20% on the follow-up CMR scan versus baseline early post-MI.

#### Non-infarct-related artery infarcts

Areas of non-infarct-artery late gadolinium enhancement were additionally classified as likely to be acute or chronic based on the presence of myocardial wall thickness (reduced (thinned) versus increased (thick/swollen) and oedema (yes/no) on T2-weighted sequences. This was done by two observers (KM and CB). T2-weighted CMR was not used to assess the area-at-risk as two sequences: a bright-blood T2-weighted ACUT2E method (acquisition for cardiac unified T2 oedema)^17^ and a T2 mapping method using an investigational prototype were used during the length of this study.

### Quantitative coronary analysis

The coronary angiograms underwent quantitative coronary analysis (QCA) of culprit and non-culprit lesions. The analyses were performed by an interventional cardiologist (BWH) who was blinded to the CMR and who had not been involved in any other aspect of the study. The QCA analyses were performed on a Centricity CA 1000 Cardiac Review 1.0 workstation (GE Healthcare, Buckinghamshire, UK).

### Statistical analysis

The primary outcome was the change in LV end-systolic volume index (mL/m^2^) revealed by CMR at follow-up versus baseline. A prioritised secondary outcome was the occurrence of myocardial infarction in the territory of a non-culprit lesion treated by preventive PCI (ie, iatrogenic myocardial infarction) (see online supplementary material methods).

#### Sample size calculation

For a between-group treatment difference of 3.0 mL/m^2^ for the within-subject change in LV end-systolic volume index at follow-up versus baseline and an SD of 4.5 mL/m^2^ then 37 subjects with evaluable data would be required in each group to refute the null hypothesis of no difference with 80% power and a two-sided test of significance level (α=0.05).

#### Data analysis

Categorical variables were expressed as number and percentage of patients. Categorical variables were analysed using a χ^2^ test, or a Fisher's exact test if any of the expected cell sizes were <5. Most continuous variables had a skewed distribution and are therefore presented as median and IQR, and analysed using Mann-Whitney statistical tests. Statistical analysis was not carried out on baseline characteristics as per the CONSORT statement recommendation.^18^

We also carried out a multivariate regression analysis model, adjusting log-corrected total infarct size between groups for MI location (anterior vs non-anterior), time to reperfusion, age of participants at presentation, thrombolysis in myocardial infarction (TIMI) score (pre-PCI), Rentrop score (pre-PCI) and diabetes mellitus status.

A p value <0.05 was taken as significant. The statistical packages used in the analysis were R V.2.15 (http://www.r-project.org) and Minitab 17 (http://www.minitab.com).

## Results

### Patient characteristics

**Figure 1:**
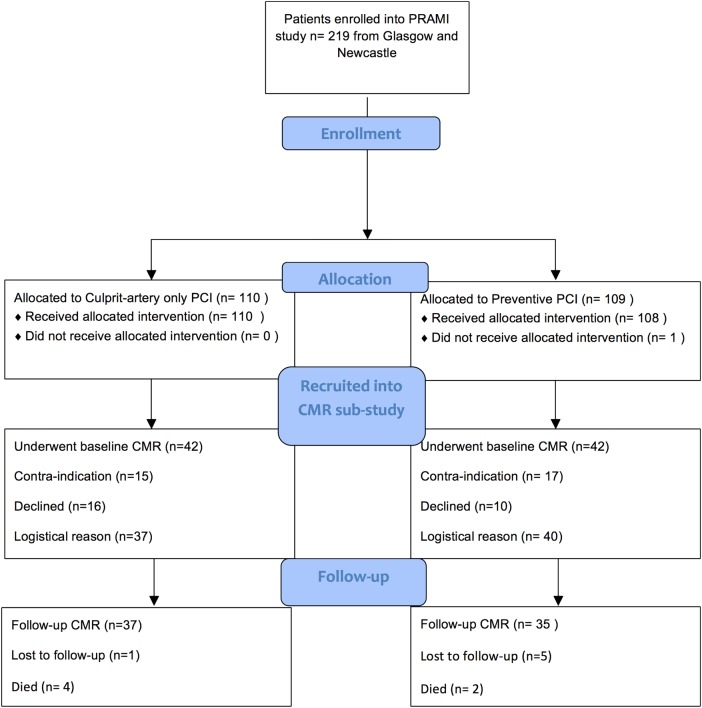
CONSORT flow diagram depicting the PRAMI CMR substudy. A total of 219 patients were enrolled in Glasgow and Newcastle and 84 of these patients gave informed consent to participate in the CMR substudy. CMR, cardiac magnetic resonance; PCI, percutaneous coronary intervention; PRAMI, preventive angioplasty in myocardial infarction.

Of 465 subjects enrolled in six sites in the PRAMI trial, 219 (47%) were enrolled in two hospitals in Glasgow and Newcastle. Of these, 84 (38%) participants (mean age 60.4 SD 11.1 years, 77% male) (table 1) underwent CMR at baseline during the first week post-MI. Figure 1 illustrates the flow diagram of the randomised participants, including the reasons for not undergoing CMR.

**Table 1:** Characteristics of the PRAMI participants and participants in the CMR substudy

Characteristics	PRAMI preventive	CMR substudy preventive	PRAMI culprit only	CMR substudy culprit only
Number of participants	234	42	231	42
Mean age (range), years	62 (32–92)	61 (38–89)	62 (33–90)	60 (39–83)
Male	177 (76)	31 (74)	186 (81)	34 (81)
Female	57 (24)	11 (26)	45 (19)	9 (21)
Medical history, n (%)				
Diabetes	35 (15)	5 (12)	48 (21)	3 (7)
Hypertension	94 (40)	10 (24)	93 (40)	11 (26)
Smoker	118 (50)	31 (74)	103 (45)	26 (62)
Previous stroke	10 (4)	0 (0)	10 (4)	0 (0)
Previous myocardial infarction	19 (8)	0 (0)	16 (7)	1 (2)
Blood pressure, mm Hg*				
Systolic	136 (26)	143 (24)	134 (26)	142 (29)
Diastolic	81 (14)	81 (13)	80 (15)	85 (17)
ST-elevation location, n (%)				
Anterior	67 (29)	10 (24)	89 (39)	23 (55)
Inferior	154 (66)	28 (67)	128 (55)	15 (36)
Lateral	10 (4)	1 (2)	14 (6)	2 (5)
Coronary arteries with stenosis, n (%)				
2	143 (61)	33 (79)	155 (67)	30 (71)
3	91 (39)	9 (21)	76 (33)	121 (29)
Infarct artery, number of stents*				
Infarct artery*	1.56 (0.75)	1.51 (0.60)	1.42 (0.70)	1.41 (0.63)
Non-infarct artery*	1.36 (0.77)	1.38 (0.55)	NA	NA
Medical therapy, n (%)				
Use of glycoprotein IIb/IIIa	178 (76)	35 (83)	176 (76)	39 (93)
Aspirin	233 (100)	42 (100)	229 (100)	42 (100)
Clopidogrel	234 (100)	42 (100)	229 (100)	42 (100)
Statin	222 (95)	40 (95)	223 (97)	39 (93)
β-blocker	207 (88)	37 (88)	210 (92)	36 (86)
ACE inhibitor or angiotensin-receptor blocker	218 (93)	39 (93)	209 (91)	36 (86)

The distribution of clinical characteristics was similar between the randomised groups (p>0.05) for all comparisons.

*Mean (SD).

CMR, cardiac magnetic resonance; PRAMI, preventive angioplasty in myocardial infarction.

The participants were evenly distributed between the randomised groups (n=42 (50%) culprit-artery-only PCI; n=42 (50%) preventive PCI). The time from symptom onset to PCI was longer in the culprit-only group (average time 330±332 min) than in the preventive PCI group (273±248 min) but this difference was not statistically significant (p=0.38). Their clinical characteristics are described in table 1.

### Angiographic findings

The Syntax scores of the randomised participants were consistent with non-complex coronary disease (table 2). The Syntax and APPROACH scores and the American Heart Association classification of lesion complexity were similar in both groups.

**Table 2:** Coronary artery plaque characteristics revealed by invasive angiography in the PRAMI participants

	Culprit-artery-only PCI	Preventive PCI	p Value
Pre-PCI*	n=42	n=42	
Time to reperfusion (minutes) median, IQR	174 (129, 413)	177 (123, 326)	0.20
Syntax score pre-PCI	17.75 (12.00, 25.75)	13.00 (10.00, 21.00)	0.09
APPROACH score (QCA) pre-PCI	50.73 (31.28, 63.00)	39.60 (27.75, 58.03)	0.09
AHA classification-simple lesions (A, B1), n (%)	18 (25%)	18 (25%)	1.00
Culprit lesions*	n=43†	n=42	
Complex lesion, n (%)	41 (95%)	42 (100%)	
Median QCA stenosis ratio (% diameter)	100.00 (65.14, 100.00)	100.00 (78.85, 100)	0.16
Lesion length (mm)	11.66 (9.48, 14.38)	13.64 (8.63, 17.72)	0.353
APPROACH score (QCA)	27.75 (18.50, 44.50)	27.50 (18.50, 28.24)	0.12
Non-culprit lesions*	n=56	n=54	
Complex lesion, n (%)	21 (38%)	31 (57%)	
Median QCA stenosis ratio (% diameter)	59.53 (51.66, 78.68)	56.31 (48.92, 65.20)	0.06
Lesion length (mm)	10.43 (7.14, 13.74)	10.48 (7.66, 15.29)	0.632
APPROACH score (QCA)	18.50 (0.00, 27.75)	15.25 (0.00, 29.70)	0.68
Post-PCI*	n=42	n=42	
Intraprocedural thrombotic events n (%)	6 (14)	9 (21)	0.754
Syntax score, post-PCI	4.00 (3.00, 7.25)	0.00 (0.00, 0.00)	<0.001
APPROACH score (QCA)	18.50 (0.00, 27.75)	0.00 (0.00, 0.00)	<0.001

*Continuous data are summarised by median (IQR); Mann-Whitney test for continuous data, χ^2^ for categorical variables.

†One patient in the culprit-only group had two culprit lesions in the same coronary artery.

AHA, American Heart Association; PCI, percutaneous coronary intervention; PRAMI, preventive angioplasty in myocardial infarction; QCA, quantitative coronary analysis.

Preventive PCI reduced the angiographic burden of disease and extent of myocardial jeopardy, as revealed by the post-PCI Syntax and APPROACH scores, respectively (table 2).

### CMR findings

At baseline, 80 (95%) of the cine imaging, and 73 (87%) of late gadolinium enhancement (LGE) imaging were of high quality. At follow-up, 68 (94%) of the cine imaging, and 68 (94%) of the LGE imaging were of high quality. At baseline 2 (2%) of the T2 weighted imaging and LGE were missing (see online supplementary table S1).

LV ejection fraction and volumes, and remodelling were similar in each of the randomised groups at baseline and follow-up (table 3; see online supplementary table S2). The timing of CMR was similar between the groups (table 3; see online supplement).

**Table 3:** Baseline and follow-up CMR

	Culprit-artery-only PCI	Preventive PCI	p Value
CMR at baseline, n=84*	n=42	n=42	
LV ejection fraction, %†	47.9 (40.3, 47.9)	48.5 (38.6, 55.8)	0.96
LV end-diastolic volume index, mL/m^2^†	64.8 (57.1, 77.4)	68.5 (54.7, 79.0)	0.86
LV end-systolic volume index, mL/m^2^†	33.5 (27.3, 47.8)	34.1 (25.5, 49.1)	0.92
Total infarct size (% LVM)*‡	n=41	n=41	
Median (IQR)	15.66 (6.18, 28.78)	14.62 (4.81, 20.10)	0.33
Mean (SD)	(18.12 (13.85))	(14.83 (11.75))	–
Time from PPCI (days), mean (range)	5 (0–32)	4 (1–14)	0.41
Infarct on LGE, n (%)	37 (90)	39 (95)	0.67
Microvascular obstruction, n (%)	10 (24)	8 (20)	0.60
Microvascular obstruction, % LV mass†	0.00 (0.00, 0.60) range (0.00:0.28)	0.00 (0.00, 0.00) range (0.00:0.15)	0.64
Infarct in non-infarct-related artery territory, n (%)	2 (5)	4 (10)	0.68
Acute infarct in non-infarct-related artery territory, n (%)	0 (0)	2 (5)	0.15
Culprit-artery infarct size (% LVM) irrespective of acute or chronic§			
Median (IQR)	15.66 (6.18, 28.78)	13.25 (3.87, 17.85)	0.16
Mean (SD)	(17.91 (13.91))	(13.55 (11.60))	–
Non-culprit-artery infarct size (% LVM)			
Mean±SD	4.34±0.79	9.70±4.41	0.11
Follow-up CMR	n=37	n=35	
Time to CMR (days), median (IQR)	210 (195, 994)	209 (186, 419)	0.42
Total infarct size (% LVM)‡			
Median (IQR)	13.43 (3.30, 22.15)	7.68 (2.10, 12.09)	0.14
Infarct on LGE, n (%)	35 (95)	32 (91)	0.66
Patients with >1 infarct, n (%)	4 (11)	5 (14)	0.73
Adverse remodelling, n (%)	4 (10)	7 (17)	0.52
LV ejection fraction, %†	51.7 (42.9, 60.2)	54.4 (49.3, 62.8)	0.23
LV end-diastolic volume index, mL/m^2^†	69.3 (59.4, 79.9)	66.1 (54.7, 73.7)	0.48
LV end-systolic volume index, mL/m^2^†	31.8 (24.4, 43.0)	30.7 (23.0, 36.3)	0.20

*One of the patients randomised to culprit-artery-only PCI had an estimated glomerular filtration rate (eGFR) <30 mL/min/1.73 m^2^ and thus IV gadolinium contrast agent was not administered. One of the patients randomised to preventive PCI could not tolerate the CMR scan, scanning was terminated after acquisition of cine sequences. % LVM (% left ventricular mass).

†Mean (SD) or median (IQR).

‡Total infarct size includes both acute and chronic infarcts.

§Acute infarct size adjusted (age, anterior MI, TIMI pre-coronary intervention, time to reperfusion, diabetes mellitus (DM), Rentrop) gives a p value of 0.735 comparing culprit-artery-only PCI with preventive PCI.

CMR, cardiac magnetic resonance; LV, left ventricular; PCI, percutaneous coronary intervention; PPCI, primary percutaneous coronary intervention.

The primary outcome (cardiac death, non-fatal MI and refractory angina) had occurred in four participants assigned to preventive PCI and in seven participants assigned to culprit-artery-only PCI (table 4).

**Table 4:** Prespecified adverse clinical outcomes in the CMR substudy participants

Characteristic	Culprit-artery-only PCI n=42 (50%)	Preventive PCI n=42 (50%)
Primary outcome*		
Death from cardiac causes, non-fatal myocardial infarction or refractory angina	7	4
Death from cardiac causes or non-fatal myocardial infarction	4	2
Death from cardiac causes	2	0
Non-fatal myocardial infarction	2	2
Refractory angina	3	2
Secondary outcomes*		
Death from non-cardiac causes	2	2
Repeat revascularisation	4	3

In line with the preventive angioplasty in myocardial infarction (PRAMI) protocol^11^ the study participants underwent standard care follow-up led by the attending physician. Routine stress testing was not performed and instead stress tests and repeat revascularisation during follow-up were clinically indicated based on a history of angina in line with contemporary guidelines.

*The follow-up interval is from randomisation to 1 December 2015.

CMR, cardiac magnetic resonance; PCI, percutaneous coronary intervention.

Infarct size and distribution revealed by late gadolinium enhancement imaging were similar in each of the randomised groups at baseline and at follow-up (table 3). Two patients did not have late gadolinium enhancement acquisitions. One of the patients randomised to culprit-artery-only PCI had an eGFR <30 mL/min/1.73 m^2^ and thus IV gadolinium contrast agent was not administered. One of the patients randomised to preventive PCI could not tolerate the CMR scan, so scanning was terminated after acquisition of the cine sequences.

On multivariate regression analysis, there was still no significant difference in infarct size between patients randomised to preventive PCI and culprit-artery-only PCI (p=0.74). Using the same model approach but with log-transformed infarct-artery infarct size instead of total infarct size, this model yielded a between-group p value of 0.95.

**Figure 2:**
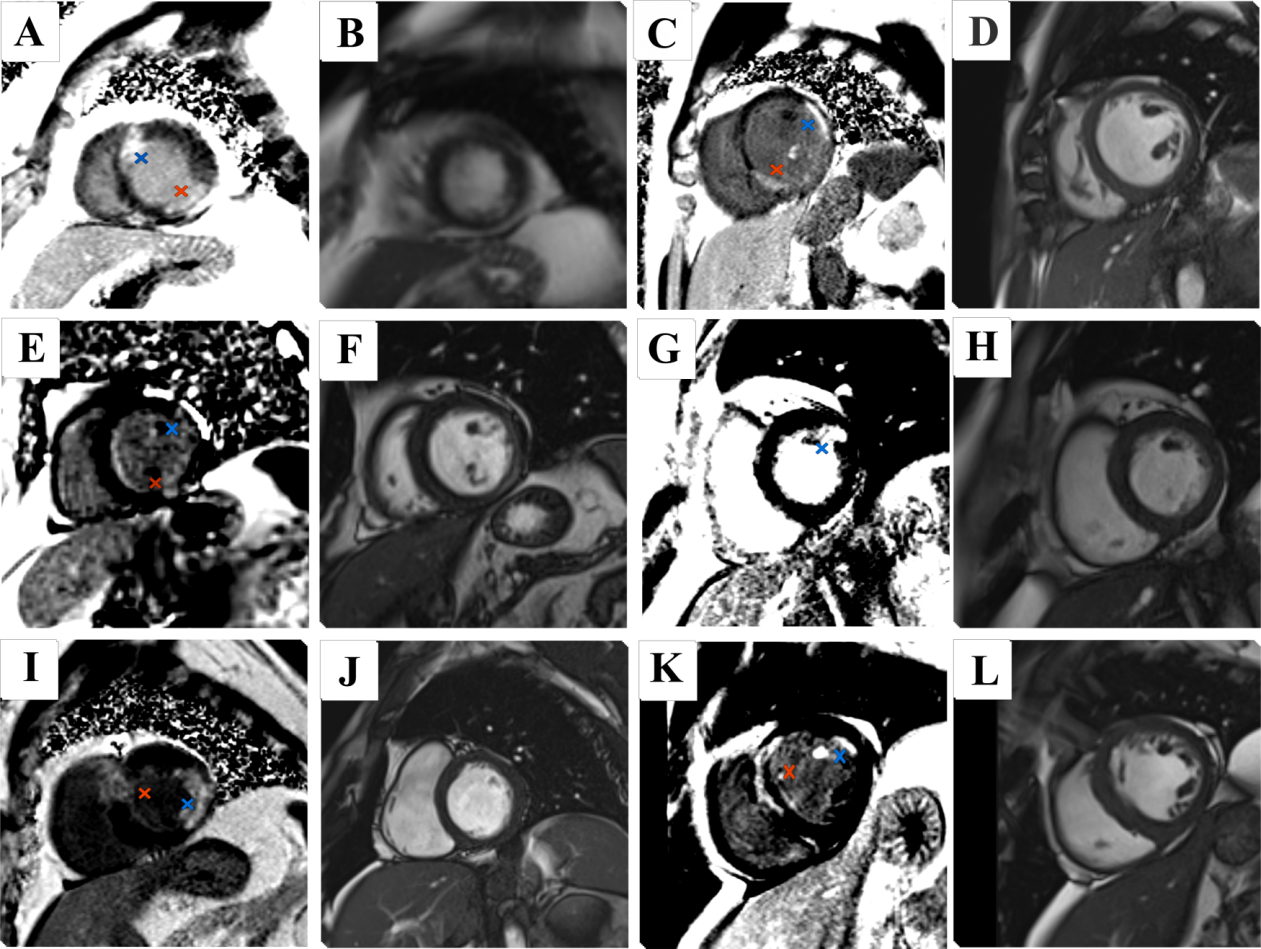
Late gadolinium enhancement imaging of preventive angioplasty in myocardial infarction (PRAMI) cardiac magnetic resonance (CMR) substudy participants depicting non-culprit-artery infarcts, with corresponding end-diastolic cine frame. Red crosses indicate culprit-artery-territory infarct; blue crosses indicate non-culprit-artery-territory infarct. Preventive percutaneous coronary intervention (PCI): (A, B) patient with lateral ST elevation on ECG underwent PCI to the circumflex and to the mid-left anterior descending coronary artery (LAD). Late enhancement revealed an additional region of scar in the anteroseptal wall. (C, D) Participant with an inferior ST-elevation myocardial infarction (STEMI) with PCI to the right coronary artery (RCA) underwent CMR which revealed an additional infarct in the anterolateral region. This patient underwent PCI to all three coronary arteries. (E, F) This patient had PCI to the RCA for an inferior STEMI and PCI to the circumflex artery. Late enhancement revealed a small focus of scarring in the basal anterolateral segment. (G, H) Inferior STEMI with PCI to the culprit RCA and additional PCI to the circumflex. Late enhancement revealed a small infarct in the basal anterolateral segment. Culprit-artery-only PCI: (I, J) participant presented with an anterior STEMI. Late gadolinium enhancement revealed additional inferolateral infarct. (K, L) Anterior STEMI, with late enhancement in the anteroseptal region and an additional area in the anterolateral segment.

On the baseline CMR scans, four patients randomised to preventive PCI had evidence of late gadolinium enhancement in the territory of a non-culprit-artery treated by PCI (table 3; figure 2). In two of these cases, this abnormality was associated with myocardial wall thinning on cine acquisitions and thus these infarcts were considered chronic. The two patients randomised to culprit-artery-only PCI who had evidence of remote zone scar did not have oedema on T2-weighted imaging, and on cine acquisitions had the myocardium was thinned. These abnormalities were considered to be chronic infarction.

## Discussion

The main findings in the PRAMI CMR substudy were that, in line with our first hypothesis, the incidence of iatrogenic myocardial infarction in the preventive PCI group was uncommon (4.8%), and appears to be lower than the incidence of non-infarct-artery MI (17%) in the population of patients who underwent CMR in the CvLPRIT trial.^14^ Infarct size was similar between groups within the first week after randomisation, even after adjustment for confounders. Second, LV volumes and ejection fraction were similar acutely and in the longer term, suggesting that differences in LV remodelling are unlikely to explain the benefits of preventive PCI observed in the PRAMI trial.

### Strengths and limitations of the current study and results

The strengths of our study include the use of multimodality CMR to assess infarct characteristics, including non-infarct-artery MI and LV remodelling.^19^ Second, the CMR substudy participants represented one-third of the PRAMI trial population in the participating centres, and their clinical characteristics were broadly similar to those of the overall trial population.^11^ As in the main trial, by chance, anterior myocardial infarction was more common in the infarct-artery-only PCI group. On the other hand, the APPROACH Lesion Score for myocardial jeopardy, and coronary plaque characteristics, were similar between the groups. The comparatively long duration of follow-up was intended to align with that of the overall trial and thus reflect longer-term changes in infarct size and LV remodelling. We also assessed clinical characteristics associated with PCI-related complications^20–22^ (table 1).

The main limitation of our study is the sample size. The study is underpowered to detect between-group differences in LV end-systolic volume (<3.0 mL/m^2^), LV ejection fraction and infarct size. We used oedema imaging qualitatively to identify areas of acute injury, however, we did not quantify the area-at-risk and myocardial salvage index, as two different T2-weighted sequences were used during the course of the study (see online supplement). CMR information on deceased patients was lacking.

### Insights into the potential benefit of preventive PCI

The LV volumes and ejection fraction in the surviving patients who completed CMR follow-up were similar in each of the groups. While a type 2 error is not discounted, these CMR results support the possibility that the benefit of the immediate preventive PCI strategy in PRAMI may be mediated by prevention of recurrent spontaneous cardiac events by immediate PCI, in line with the main results of PRAMI^11^ and CvLPRIT.^12^ Our results are uniformly consistent with those observed in the larger CvLPRIT CMR substudy^14^ that involved 205 randomised participants. In that study, as in our own, there were no between-group difference in LV volumes and ejection fraction or infarct size, on baseline and follow-up CMR.^23^ ^24^ Missing CMR data from deceased patients are a relevant gap since one mechanism of benefit of preventive PCI may be prevention of fatal MI and cardiac death.

### Importance of the timing of enrolment and randomisation: before versus after culprit-artery PCI and relevance to clinical practice

Based on our angiographic analysis, the non-culprit lesions had low-risk Syntax scores and 47% had non-complex characteristics (table 2), implying selection of lower risk lesions for immediate preventive PCI. In PRAMI, participant eligibility was based on the presence of non-culprit lesions that were deemed by the cardiologist to be amenable to PCI and therefore enrolment after successful culprit-artery PCI was at operator discretion. In other words, preventive PCI was performed in patients in whom the operator believed additional immediate PCI would be feasible, safe and successful. The 4.8% incidence of iatrogenic MI in the preventive PCI group in our PRAMI CMR study was lower than in other reports^23^ ^24^ (eg, 12% incidence of iatrogenic MI in the CvLPRIT CMR substudy). Importantly, the study design of the PRAMI^11^ and CvLPRIT^12^ trials differed. Based on our CMR findings, PRAMI has a low incidence of procedure-related MI, unlike in CvLPRIT. This difference may be explained by randomisation before culprit-artery PCI in CvLPRIT and so operators being required by protocol to revascularise lesions that they might otherwise not have treated on technical grounds (be the procedure acute or staged within the index admission), implying higher risk procedures that inevitably would be associated with a higher risk of complications and procedure-related MI. By contrast in PRAMI, the protocol invoked the trial intervention after successful culprit-artery PCI and only in patients who had an artery amenable to PCI (and that was appropriately interpreted to be ‘at that time’). So in PRAMI, there was flexibility to treat lesions (or not) whereas in CvLPRIT, the operator did not have that choice. This is further reflected in the lower incidence of microvascular obstruction in our study group (21%) when compared with other imaging studies in STEMI.^14^ ^25^ ^26^ This would suggest that the patient group was lower risk, with an associated lower event rate. The comparatively low incidence of microvascular obstruction in our study population may be one contributing factor for the incidence of adverse health outcomes, since microvascular obstruction is a complication post-STEMI that portends an adverse prognosis.^27^ ^28^

From the perspective of clinical translation to every day practice, this difference lends support for clinicians to focus on non-infarct-related artery lesions that are amenable to preventive PCI and with the decision taking place in the cardiac catheter laboratory immediately after successful culprit-artery PCI. On the other hand, patients with higher risk clinical characteristics or more complex non-infarct-artery disease could be deferred for staged inpatient revascularisation. We think this strategy more closely represents how preventive PCI might be adopted by clinicians in real-life clinical practice.

### Clinical relevance of the CMR findings: contemporary guidelines and current trials

The guidelines of the European Society of Cardiology on myocardial revascularisation state that ‘Immediate revascularization of significant non-culprit lesions during the same procedure as primary PCI of the culprit vessel may be considered in selected patients’ (IIb recommendation, Level of Evidence: B).^8^ The North American guidelines now have a similar IIb recommendation.^10^

There are two other clinical trials comparing culprit-only PCI to complete revascularisation in patients with STEMI and multi-vessel disease (MVD). The complete versus culprit-only revascularisation to treat multivessel disease after primary PCI for STEMI (COMPLETE; NCT01740479; estimated sample size 3900 patients)^29^ is designed to assess whether a strategy of complete revascularisation involving staged PCI of all suitable non-infarct-related artery lesions is superior to a strategy of culprit lesion-only revascularisation in reducing the composite outcome of cardiovascular death or MI in patients with MVD who have undergone successful culprit lesion primary PCI for STEMI. The comparison between fractional flow reserve (FFR)-guided revascularisation versus conventional strategy in ACUTE STEMI patients with MVD (COMPARE-ACUTE; NCT01399736; estimated sample size 885)^30^ has broadly similar objectives as COMPLETE, but is a smaller in scale. These trials should provide meaningful evidence to inform clinicians and practice guidelines on the optimal management of STEMI patients with MVD.

## Limitations

The PRAMI CMR substudy involved a limited proportion of the total number of participants in the main trial PRAMI trial, reflecting the fact that two of the six centres participated in the CMR study. Further studies are warranted. The distributions of some of the characteristics of participants in the substudy, for example, anterior infarct location, departs from those of the main trial population. The number of clinical events in the substudy is lower than in the main trial. Because of changes in the availability of oedema imaging methods, two T2-weighted CMR methods were used during the lifetime of the project, making quantitative assessment of area-at-risk and myocardial salvage not feasible. Two of the patients did not have late gadolinium CMR imaging (one in the culprit-only PCI group and one in the preventive PCI group). Measurement of cardiac biomarkers (eg, serial troponin testing) was not part of the PRAMI trial protocol. There was heterogeneity in the duration of follow-up due to logistical reasons.

## Conclusions

We have performed an exploratory CMR substudy in the PRAMI participants. We conclude that the benefit from a preventive PCI strategy was unrelated to infarct size or LV remodelling, although the strength of this conclusion is tempered by the fact that study was underpowered to detect differences in these parameters. Further studies are required.

**Key messages**

**What is already known on this subject?**

- Percutaneous coronary intervention (PCI) in patients with acute ST-elevation myocardial infarction (STEMI) and multivessel coronary disease is associated with improved clinical outcomes. However, doubt remains about the benefit of preventative PCI of other coronary arteries in addition to culprit vessel revascularisation.

**What might this study add?**

- In this preventive angioplasty in myocardial infarction (PRAMI) cardiac magnetic resonance substudy, compared with culprit-only percutaneous coronary intervention (PCI), immediate preventive PCI of additional vessels was associated with a low incidence of iatrogenic myocardial infarction (n, % (culprit-artery-only PCI vs preventive PCI), 0 (0) vs 2 (5), p=0.15) with no difference at follow-up in left ventricular (LV) ejection fraction or LV volumes.

**How might this impact on clinical practice?**

- A routine strategy of immediate preventive percutaneous coronary intervention is a reasonable approach in selected patients with acute ST-elevation myocardial infarction and multivessel coronary disease.
