# Supplementary material for: Infarct size and left ventricular remodelling after preventive percutaneous coronary intervention
Source: Heart. 2016 Aug 8;102(24):1980–7. doi: 10.1136/heartjnl-2015-308660 (PMC5256395; doi:10.1136/heartjnl-2015-308660)
Supplement: Supplementary data [file heartjnl-2015-308660supp.pdf]

Infarct size and left ventricular remodelling after preventive percutaneous coronary intervention.

Supplementary Methods

Study Design

Treatment assignment was determined by randomisation in the cardiac catheter laboratory in patients with TIMI grade 3 coronary flow in the culprit artery following primary PCI. Patients with one or more non-culprit artery stenoses ≥ 50% of the reference vessel diameter by visual assessment were invited to participate and following informed consent, were randomly allocated to either culprit-artery only PCI or multivessel preventive PCI. The randomisation strategy was stratified by site.

Any patient with subsequent symptoms of angina that were not controlled with the use of medical therapy was required to undergo an objective assessment of ischaemia to secure a diagnosis of refractory angina. Further PCI for angina was performed only in cases of refractory angina.

Cardiac magnetic resonance imaging-supplementary information.

Following the initiation of the Golden Jubilee National Hospital as a site in the PRAMI trial (February 2009), a protocol amendment for CMR was approved by the UK National Research Ethics Service. We therefore performed a prospective pre-specified CMR sub-study involving the trial participants who were randomised at the Golden Jubilee National Hospital, Clydebank, and at the Freeman Hospital,

Newcastle upon Tyne, between May 2009 and December 2012. The duration of follow-up and timing of repeat CMR was intended to reflect the longer-term follow-up in the main trial.

PRAMI participants who were eligible for CMR were prospectively invited by the clinical research staff to participate and all of the participants provided written informed consent. In January 2013, enrolment in the PRAMI trial was stopped on the recommendation of the Data and Safety Monitoring Committee because of highly statistically significant between-group difference in the incidence of the primary outcome in favour of preventive PCI and further enrolment in the trial would not result in a change in this result.

Participants and Eligibility

The eligibility criteria for the CMR study were:

Inclusion criteria

1. Informed consent for the PRAMI trial.

Exclusion criteria

1. Implanted medical devices: e.g. pacemakers, cochlear implants.

2. Intra-cranial foreign bodies.

3. Cardiogenic shock.

Patients with a severe reduction in renal function (i.e. a glomerular filtration rate <30 mL/min/1.73m^{2}) were eligible for CMR although administration of the gadolinium

contrast agent was not permitted. A safety checklist was completed prior to the CMR examination.

CMR acquisition

The long-term duration of CMR follow-up was intended to reflect the duration of follow-up in the main trial, and so provide longer term information on infarct size (including de novo infarction) and LV function and remodelling that might be associated with each of the randomised treatment strategies.

CMR methods

Cardiac MR image acquisition

CMR was performed on Siemens MAGNETOM Avanto (Erlangen, Germany) 1.5-Tesla scanners with a 12-element phased array cardiac surface coil.

LV dimensions were assessed using retro-gated balanced steady state free precession cinematographic breath-hold sequences. The in-plane resolution was approximately 2 mm (26 μl/voxel) and the temporal resolution was approximately 40 ms within the cardiac cycle. The heart was imaged in multiple parallel short-axis (SAX) planes 8-mm thick separated by 2-mm gaps, as well as in the 2-chamber, 3-chamber, and 4-chamber long-axis views.

Myocardial infarction was imaged using turbo fast low-angle shot (Turbo-FLASH) with or without segmented phase-sensitive inversion recovery (PSIR). Infarction was assessed 15 minutes after intravenous injection of 0.15 mmol/kg of gadoterate

meglumine (Gd2+-DOTA, Dotarem, Guebert S.A.). Imaging parameters were as previously described (1).

Infarct definition and size

Infarction was defined by the occurrence of myocardial late gadolinium enhancement revealed in orthogonal planes including phase swap acquisitions as appropriate to rule out artefact. Acute infarction was defined as a territory of infarct scar that was not associated with myocardial wall thinning on cinematographic sequences. The myocardial mass of late gadolinium (grams) was quantified using computer assisted planimetry and the territory of infarction was delineated using a signal intensity threshold of >5 standard deviations above a remote reference region and expressed as a percentage of total LV mass. The occurrence of infarction in non-culprit artery territories at baseline and during longer-term follow-up was assessed by late gadolinium enhancement imaging, and in addition to regional wall motion, features consistent with acute myocardial infarction i.e. myocardial oedema revealed by T2-weighted CMR, or chronic myocardial infarction i.e. wall thinning, were also assessed. Infarct regions with evidence of microvascular obstruction (MVO) were included within the infarct area and the area of MVO was assessed separately and also as a percentage of total LV mass. LGE sequences were reviewed by K.M. and C.B. (as a senior observer with > 10 years CMR experience) to identify non-IRA infarcts.

Adverse remodelling

Adverse remodelling was defined as an increase in LV end-diastolic volume and end systolic volume ≥ 20% on the follow-up CMR scan versus baseline early post-MI.

Myocardial oedema

T2-weighted CMR was used to assess for the presence or absence of myocardial oedema involving a full stack of short axis LV scans to cover the whole of the LV. Two T2-weighted sequences were used to assess myocardial oedema, the bright-blood T2-weighted ACUT2E method (Acquisition for Cardiac Unified T2 Edema) (2). T2 mapping was acquired using an investigational prototype T2-prepared (T2P) balanced steady state free precession sequence in 13 of the patients.

Typical imaging parameters were as previously described (1) Myocardial tissue with a signal intensity at least 2 SD above the mean signal obtained in the remote non-infarcted myocardium was considered hyperintense and consistent with oedema. Hyperintense zones were assessed by a cardiologist (K.M.) who was blinded to the angiographic data. The presence of oedema in association with late gadolinium enhancement was taken supporting evidence of acute infarction.

Quantitative Coronary Analysis-supplementary information

The minimum vessel diameter used in the analysis was 2 mm, and a minimum stenosis threshold used in QCA analysis was 50%. Intra-procedural thrombotic events were classified as per published guidelines (3). Syntax scores were calculated before and after PCI using version 2.11 of the online Syntax Score Calculator. The Alberta Provincial Project for Outcome Assessment in Coronary Heart Disease (APPROACH) score was used to describe the percentage of myocardium at risk based on visual and QCA estimation of stenosis ≥50% and before and after PCI (4). Coronary angiograms were assessed for the presence of chronic total occlusions (CTO) and TIMI flow grade was assessed before and after PCI. The prognosis in

patients undergoing PCI is known to relate to the number of complex plaques as defined by pre-specified criteria (5). In this study lesions were considered complex if they had at least 50% visually assessed diameter stenosis associated with at least two of the following;

- A filling defect
- Clear evidence of thrombus,
- Plaque ulceration
- Plaque irregularity
- TIMI flow <3
- Involvement of a bifurcation lesion
- Angiographic evidence of heavy calcification

Results

CMR findings- supplementary results.

There was no difference in the timing of PCI between both arms at baseline (p= 0.432) or follow-up (p=0.538).

On follow-up imaging, there were an additional 2 (4.8%) new cases of non-infarct artery related late gadolinium enhancement in the culprit-artery-only arm. In these cases, retrospective review of the coronary angiograms revealed coronary artery lesions >50% stenosis severity that, according to the PRAMI protocol, would have

been treated immediately by preventive PCI at the time of the index procedure if the patient had been randomised to that group. One of these patients was treated with PCI 3 months post-randomisation and the new infarction in this territory was consistent with peri-procedural infarction.

LV ejection fraction, LV volumes, and the proportion of patients with adverse remodelling were similar between groups, ($p > 0.05$) (Table 3).

Six (7%) participants (1 from the culprit-artery only group and 5 from the preventive PCI group) did not attend for the follow-up CMR scan, and 6 patients died, including 2 from cardiac causes (Table 4). Follow-up was from randomization up till 31st December 2015. The median (interquartile range) follow-up time was 59.1 months (51.5, 67.3) for the culprit-artery only group, and 57.1 months (43.1, 66.3) for the preventive PCI group.

References.

1. Payne AR, Berry C, Doolin O, McEntegart M, Petrie MC, Lindsay MM, et al. Microvascular Resistance Predicts Myocardial Salvage and Infarct Characteristics in ST-Elevation Myocardial Infarction. J Am Heart Assoc. 2012 Aug;1(4):e002246.
2. Aletras AH, Kellman P, Derbyshire JA, Arai AE. ACUT2E TSE-SSFP: a hybrid method for T2-weighted imaging of edema in the heart. Magn Reson Med Off J Soc Magn Reson Med Soc Magn Reson Med. 2008 Feb;59(2):229–35.
3. McEntegart MB, Kirtane AJ, Cristea E, Brener S, Mehran R, Fahy M, et al. Intraprocedural thrombotic events during percutaneous coronary intervention in patients with non-ST-segment elevation acute coronary syndromes are associated with adverse outcomes: analysis from the ACUITY (Acute Catheterization and Urgent Intervention Triage Strategy) trial. J Am Coll Cardiol. 2012 May 15;59(20):1745–51.
4. Ortiz-Pérez JT, Meyers SN, Lee DC, Kansal P, Klocke FJ, Holly TA, et al. Angiographic estimates of myocardium at risk during acute myocardial infarction: validation study using cardiac magnetic resonance imaging. Eur Heart J. 2007 Jul;28(14):1750–8.
5. Goldstein JA, Demetriou D, Grines CL, Pica M, Shoukfeh M, O’Neill WW. Multiple complex coronary plaques in patients with acute myocardial infarction. N Engl J Med. 2000 Sep 28;343(13):915–22.

Supplementary Table 1: Image Quality Scoring

Baseline CMR	Cine	T2-weighted	LGE
n=84			
High quality	80 (95%)	79 (94%)	73 (87%)
Adequate	4 (5%)	3 (4%)	9 (11%)
Non-diagnostic	0 (0%)	0 (0%)	0 (0%)
Missing data	0(0%)	2 (2%)	2 (2%)
**Follow-up CMR**	**Cine**	**T2-weighted**	**LGE**
n=72			
High quality	68 (94%)	58 (81%)	68 (94%)
Adequate	4 (6%)	3 (4%)	4 (6%)
Non-diagnostic	0 (0%)	0 (0%)	0 (0%)
Missing data	0 (0%)	11 (15%)	0 (0%)
High Quality	Well-defined endo- and epi-cardial borders (i.e. good ECG gating)<br>No flow artefact<br>No SSFP artefact within myocardium<br>No ghosting due to patient breathing		
Adequate	One or more of the following are present:<br>Slight blurring of endo- and epi-cardial borders<br>Slight artefact due to flow within blood pool but not affecting myocardium<br>SSFP artefact just within myocardium<br>Slight ghosting due to patient breathing		
Non-diagnostic	One of more of the following are present:		

	Substantial blurring of the endo- and epi-cardial borders due to poor ECG gating
	Substantial flow artefact within myocardium
	Substantial SSFP artefact obscuring myocardium
	Substantial ghosting due to patient breathing

Supplementary Table 2. Within subject change in LV end-diastolic volume index, and LV end-systolic volume index.

Follow-up CMR	n = 37 (50%)	n = 35 (50%)	p value
Within subject Δ LV end-diastolic volume index			
Median (IQR)	-1.40 (-15.20, 6.25)	1.05 (12.57)	0.19
Mean (SD)	-3.85 (14.34)	0.43 (12.57)	-
Within subject Δ LV end-systolic volume index			
Median (IQR)	-5.40 (-13.90, 1.00)	-2.20 (-7.78, 9.62)	0.10
Mean (SD)	-6.57 (10.95)	-3.12 (10.28)	-

IQR- interquartile range. SD- standard deviation
